# Supplementary material for: Predicting response to enzalutamide and abiraterone in metastatic prostate cancer using whole-omics machine learning
Source: Nat Commun. 2023 Apr 8;14:1968. doi: 10.1038/s41467-023-37647-x (PMC10082805; doi:10.1038/s41467-023-37647-x)
Supplement: Supplementary file 1 — Supplementary Information [file 41467_2023_37647_MOESM1_ESM.pdf]

Supplementary Information

Table of contents

Supplementary Figure 1	Genomic differences between good and poor responders within the discovery training cohort
Supplementary Figure 2	Gene-set enrichment analysis and AR-V7 expression
Supplementary Figure 3	Overlap predicted labels by genomics and transcriptomics model
Supplementary Figure 4	Internal validation of clinical variables-only model
Supplementary Figure 5	Performance of clinicogenomics model in similarly pre-treated subgroups in the internal validation cohort
Supplementary Figure 6	Performance of WGS-only and WTS-only models in the internal validation cohort
Supplementary Figure 7	Survival analysis of predictors from the clinicogenomics models in the internal cohort
Supplementary Figure 8	External validation genomics-only and transcriptomics-only model
Supplementary Figure 9	Importance of individual features to clinicogenomics model
Supplementary Figure 10	Distribution of individual genomic features in cohorts
Supplementary Figure 11	Evaluation of different dimensionality reduction techniques in LOOCV
Supplementary Figure 12	Comparison non-filtered and filtered transcriptomics model for external validation
Source Data	Overview of included patients and data presented in figures (See Excel file)

Supplementary Figures

Suppl. Figure 1 - Genomic differences between good and poor responders within the discovery training cohort.

a) **Tumor mutational burden (TMB) categorized per responder category.** Boxplot (with median displayed) with individual data-points depicting the number of genomic mutations, averaged per megabase over the entire genome (TMB) per sample and categorized per responder category (poor responders in orange, good responders in green). Y-axis is shown in log_{10}-scale. The median, interquartile range (IQR), and 1.5x the IQR are represented by a solid black line, box, and whiskers, respectively. Statistical significance was tested using a two-sided Mann-Whitney U test with Benjamini-Hochberg correction with the *q*-value depicted on top, using the discovery training cohort (n = 79, q < 0.001).

b) **Total number of structural variants categorized per responder category.** Same as **a**), q = 0.0015.

c) **Total number of tandem duplications categorized per responder category.** Same as **a**), q = 0.02.

d) **Total number of deletions categorized per responder category.** Same as **a**), q = 0.005.

e) **Overview of chromosomal arm aneuploidies.** Per arm, the chromosomal copy-number (as determined by GISTIC2) are shown as Z-scores within the heatmap. The upper tracks display the generalized treatment, generalized biopsy site and responder class, respectively. No statistically significant results were observed using a two-sided Mann-Whitney U test with Benjamini-Hochberg correction.

f) **Mutational frequency of previously-associated ARSI-related genes.** Frequency of samples with a somatic aberration (mutation, copy-number alteration or structural variant) per responder group. Statistical significance was tested using a two-sided Fisher's Exact test with multiple testing correction (Benjamini-Hochberg).

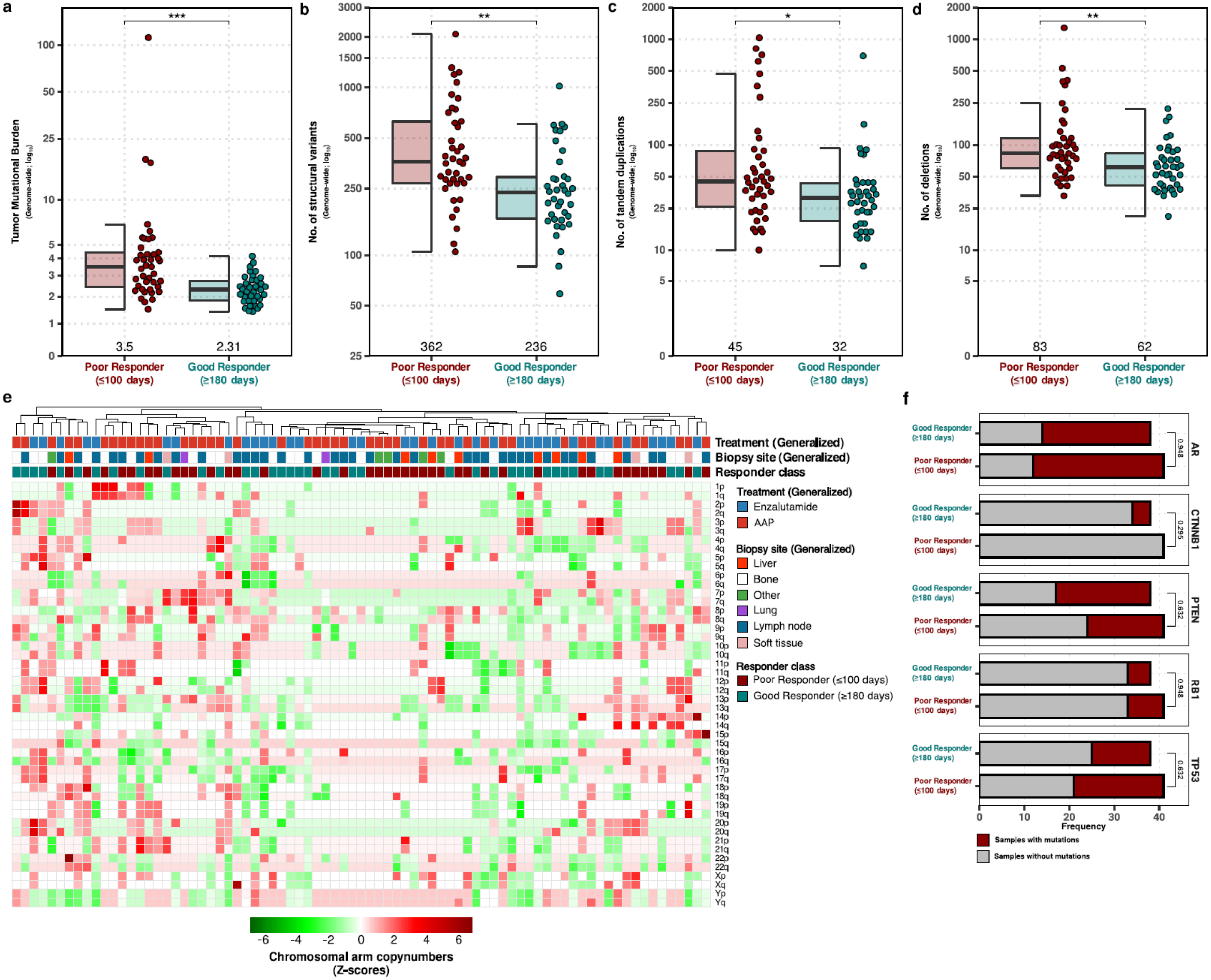
a
Tumor Mutational Burden (Genome-wide; log10)
***
3.5
2.31
Poor Responder (≤100 days)
Good Responder (≥180 days)
b
No. of structural variants (Genome-wide; log10)
**
362
236
c
No. of tandem duplications (Genome-wide; log10)
*
45
32
d
No. of deletions (Genome-wide; log10)
**
83
62
e
Treatment (Generalized)
Biopsy site (Generalized)
Responder class
Treatment (Generalized)
Enzalutamide
AAP
Biopsy site (Generalized)
Liver
Bone
Other
Lung
Lymph node
Soft tissue
Responder class
Poor Responder (≤100 days)
Good Responder (≥180 days)
Chromosomal arm copynumbers (Z-scores)
f
AR
CTNNB1
PTEN
RB1
TP53
0.948
0.295
0.632
0.948
0.632
Frequency
Samples with mutations
Samples without mutations

Suppl. Figure 2 - Gene-set enrichment analysis and AR-V7 expression.

- **a)** Normalized Enrichment Scores (NES) for significantly enriched gene-sets from the Hallmark (H) and WikiPathways (W) resources between poor vs. good responders within the internal WTS training dataset.
- **b)** Percentage spliced in (PSI) of AR-V7, relative to wild-type AR expression, for each of the responder classes. Boxplots with individual data-points are depicted with the median, interquartile range (IQR), and 1.5x the IQR represented by a solid black line, box, and whiskers, respectively. No statistically significant results were observed using a two-sided Mann-Whitney U test with Benjamini-Hochberg correction.

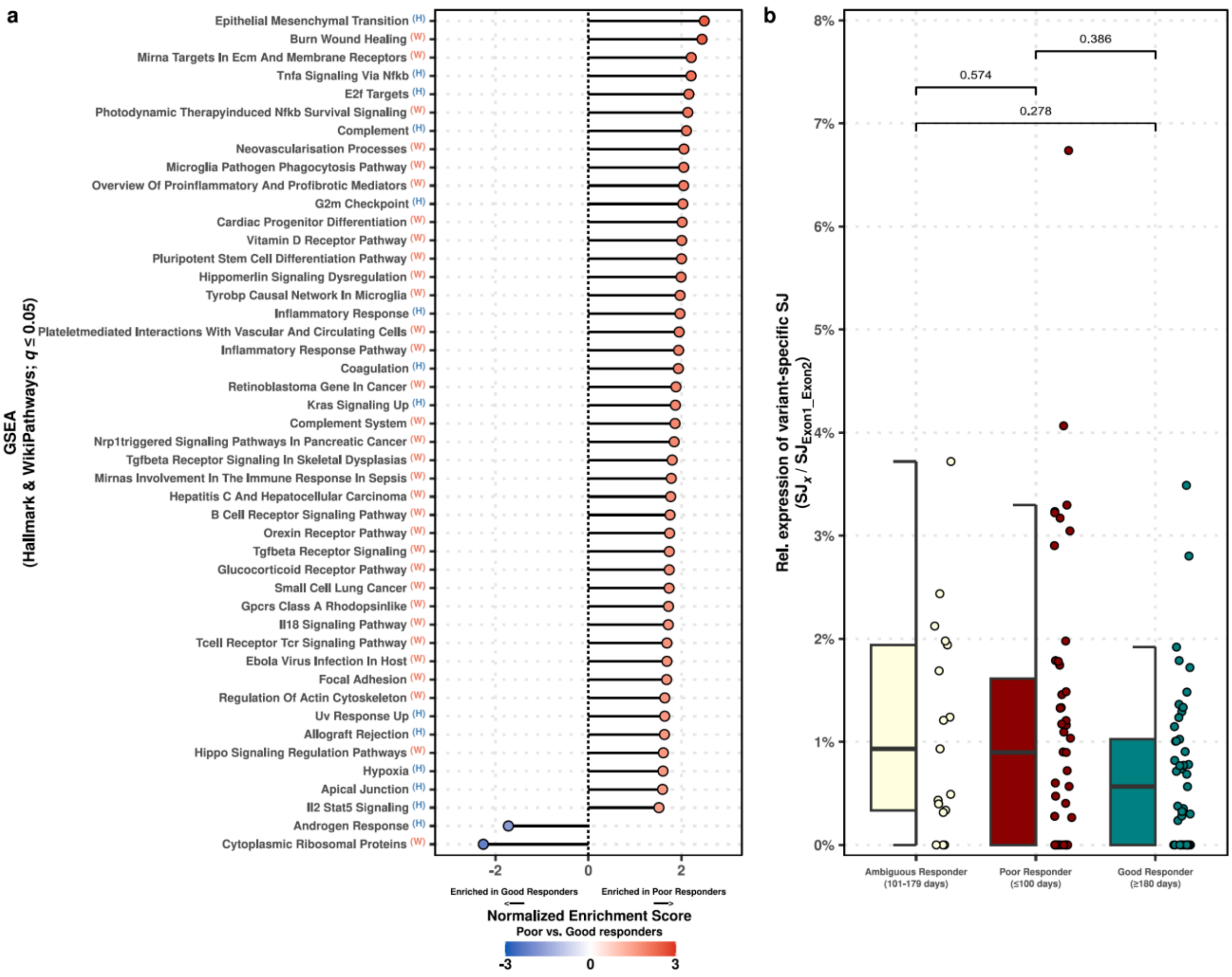

Suppl. Figure 3 - Overlap predicted labels by genomics and transcriptomics model

Venn diagrams, showing the overlap in predictions by the genomics-only and the best transcriptomics-only model (40 ICs). True positives represent correctly predicted good responders, while true negatives represent correctly predicted poor responders.

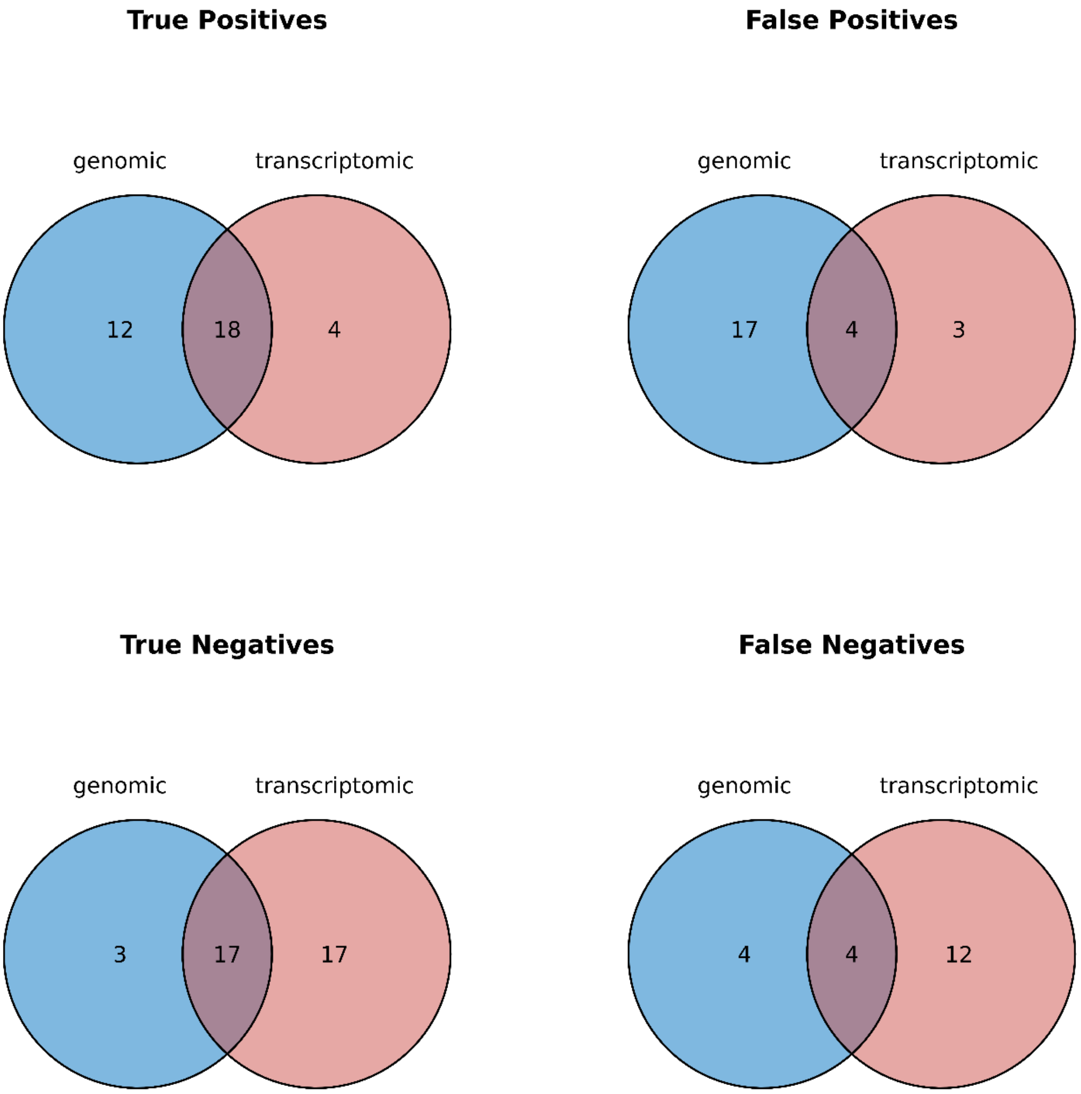

Suppl. Figure 4 - Internal validation of clinical variables-only model

Descriptive summary of clinical variables-only models on the internal validation cohort.

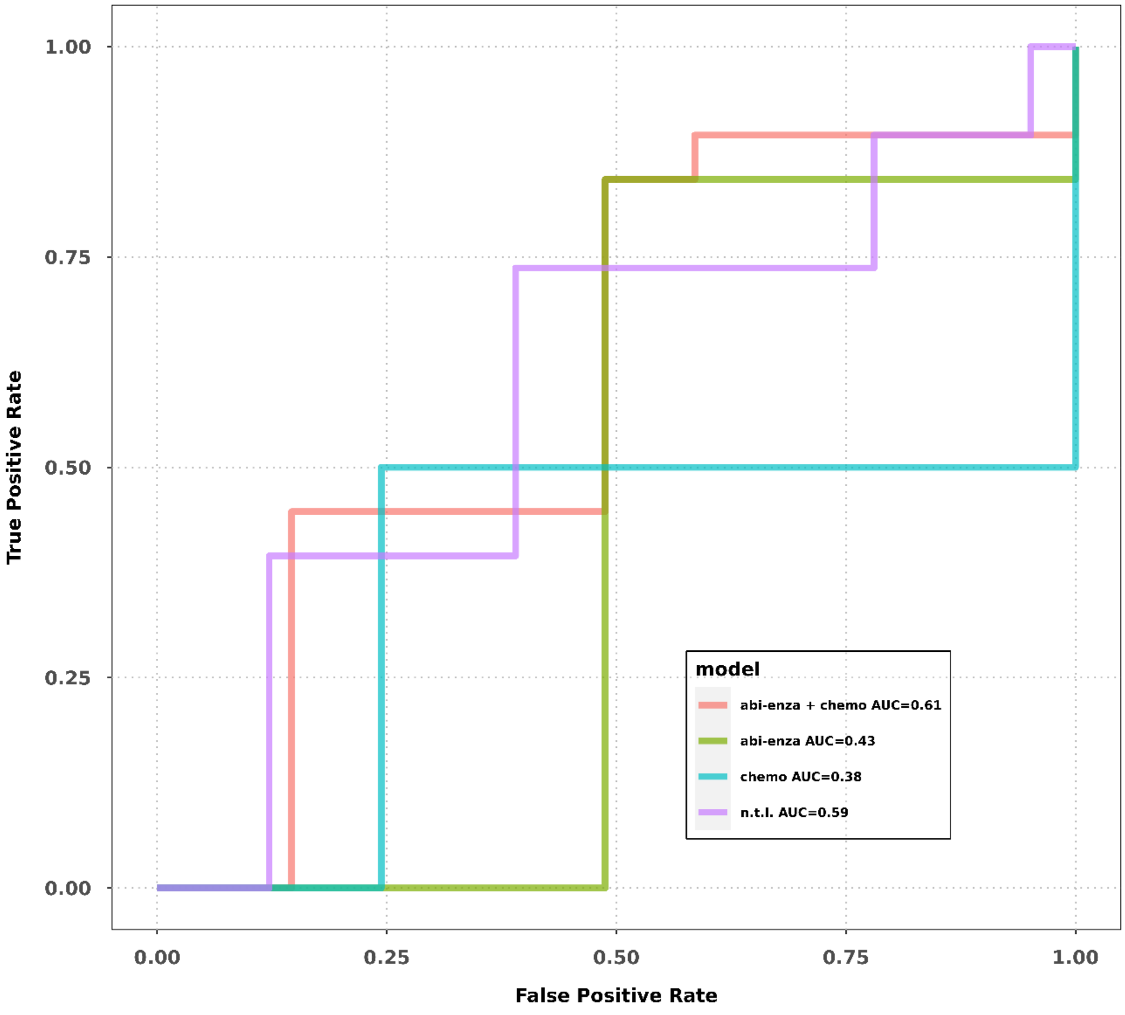

Suppl. Figure 5 - Performance of clinicogenomics model in similarly pre-treated subgroups in the internal validation cohort

Subgroups were chosen based on significant differences in prior treatment between true good and poor responders (**Table 1**). Survival analysis using ARSI-treatment duration (in days) and whether patients were still currently receiving ARSI (event) using the three-group scheme of poor, good and ambiguous predictors from the final clinicogenomics models is shown. Differences were tested using a log-rank test with an additional pairwise log-rank test between each pairwise group. Median OS with 95% confidence intervals are shown per strata. (**a**) Survival analysis on patients who received 0-1 prior systemic therapies (*n* = 42). **(b)** same as **a**, but for patients with 2+ prior systemic therapies (*n* = 34). **(c)** Same as **a**, but for patients with prior enzalutamide treatment (*n* = 60). **(d)** Same as **a**, but for patients without prior enzalutamide treatment (*n* = 16)**.**

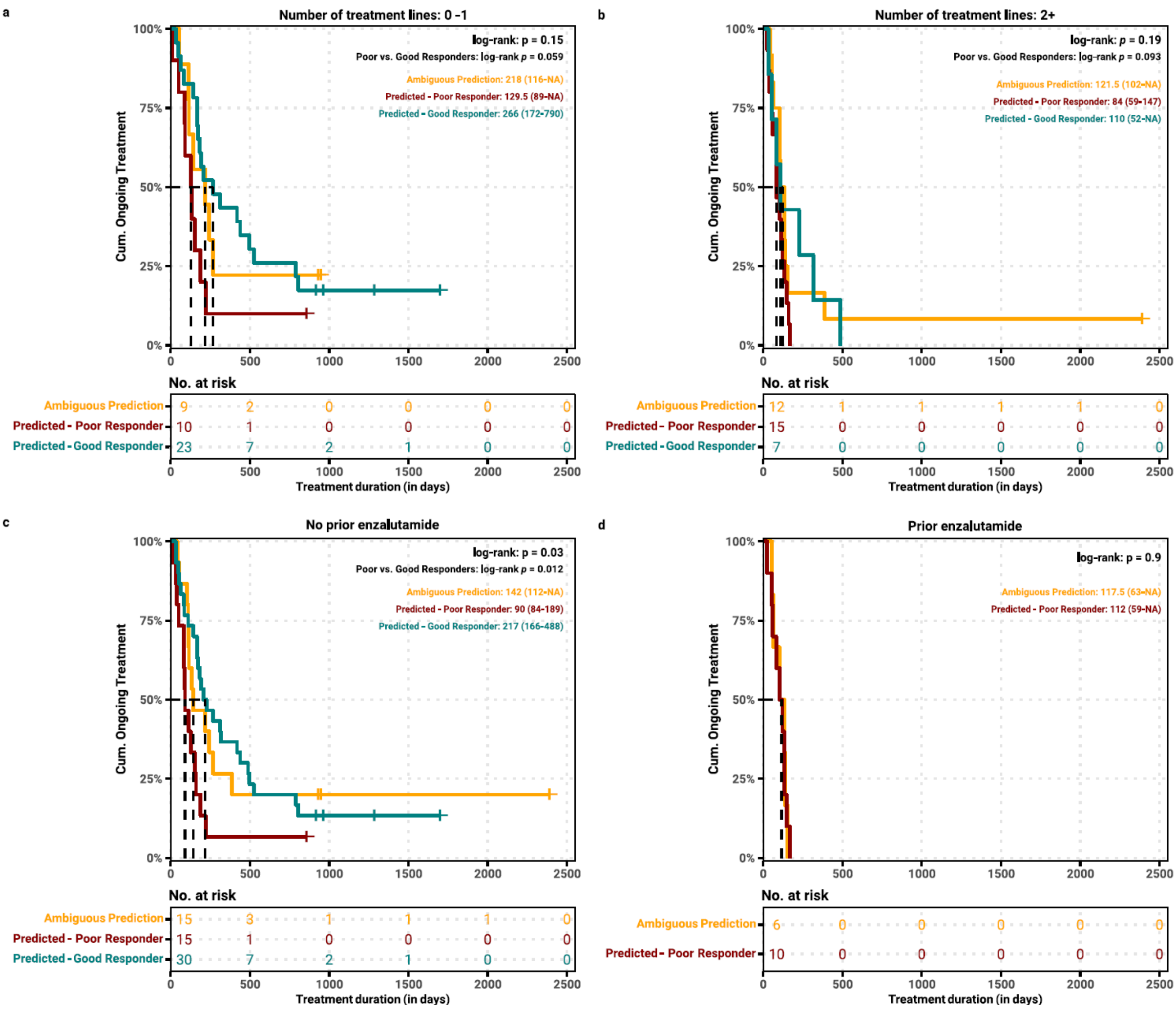

Suppl. Figure 6 - Performance of WGS-only and WTS-only models in the internal validation cohort.

- **a)** Survival analysis using ARSI-treatment duration (in days) and whether patients were still currently receiving ARSI (event) using the three-group scheme of poor, good and ambiguous predictors for the WGS-only classification model on the internal validation cohort (*n* = 76). Differences were tested using a log-rank test with an additional pairwise log-rank test between each pairwise group.
- **b)** Same as **a),** but for the WGS + prior ARSI model.
- **c)** Same as **a),** but for the WTS (on 40 ICs) model on internal validation samples with matching WTS (*n* = 34).
- **d)** Same as **a),** but for the WTS (on 40 ICs) + prior ARSI model on internal validation samples with matching WTS (*n* = 34).
- **e)** Same as **a),** but for the WGS + WTS (on 40 ICs) + prior ARSI model on internal validation samples with matching WTS (*n* = 34).
- **f)** Same as **a),** but for the WGS + WTS (on 40 ICs) + prior ARSI model on internal validation samples with matching WTS (*n* = 34).

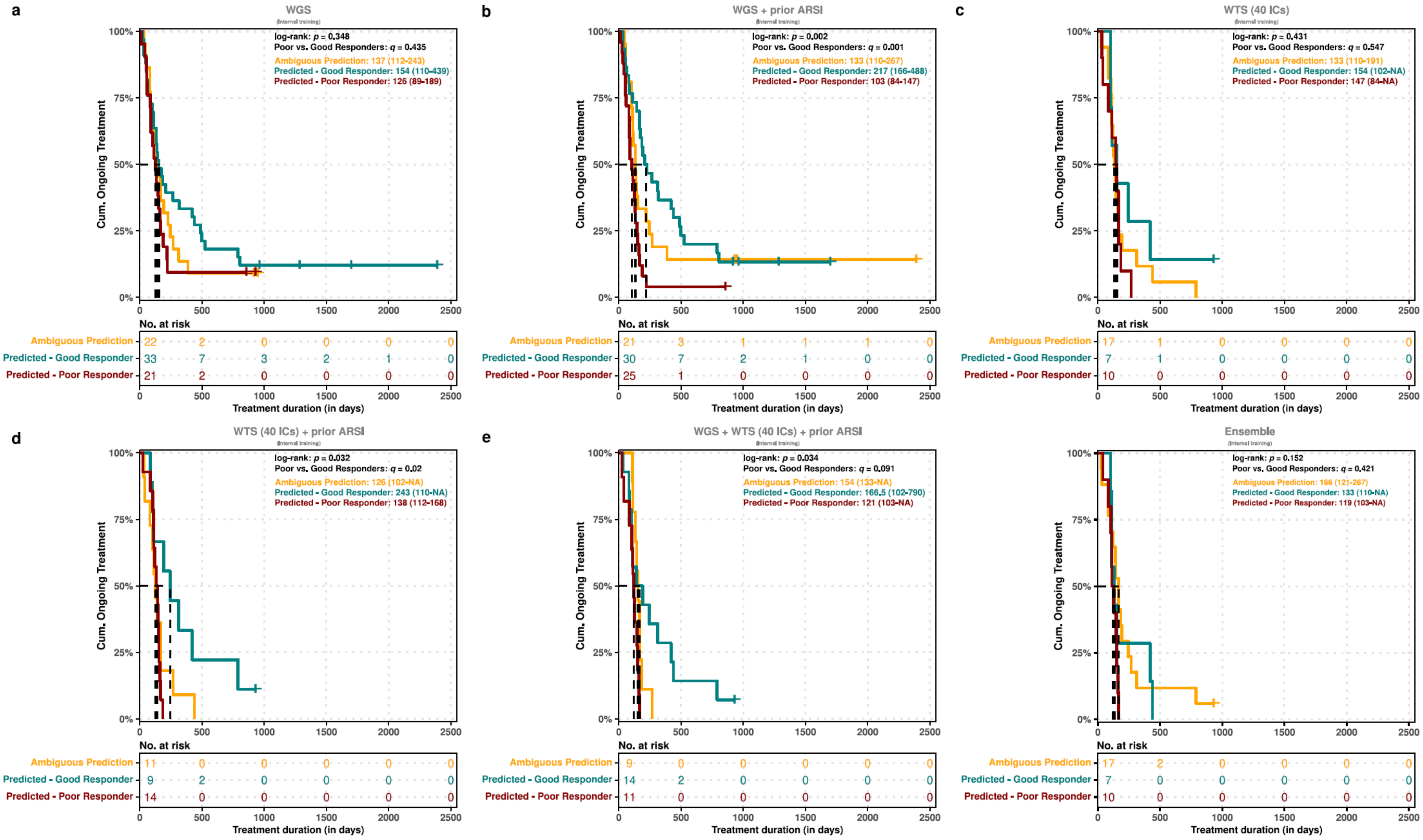

a
WGS
(Internal training)
log-rank: p = 0.348
Poor vs. Good Responders: q = 0.435
Ambiguous Prediction: 137 (112-243)
Predicted - Good Responder: 154 (110-439)
Predicted - Poor Responder: 126 (89-189)
b
WGS + prior ARSI
(Internal training)
log-rank: p = 0.002
Poor vs. Good Responders: q = 0.001
Ambiguous Prediction: 133 (110-267)
Predicted - Good Responder: 217 (166-488)
Predicted - Poor Responder: 103 (84-147)
c
WTS (40 ICs)
(Internal training)
log-rank: p = 0.431
Poor vs. Good Responders: q = 0.547
Ambiguous Prediction: 133 (110-191)
Predicted - Good Responder: 154 (102-NA)
Predicted - Poor Responder: 147 (84-NA)
d
WTS (40 ICs) + prior ARSI
(Internal training)
log-rank: p = 0.032
Poor vs. Good Responders: q = 0.02
Ambiguous Prediction: 126 (102-NA)
Predicted - Good Responder: 243 (110-NA)
Predicted - Poor Responder: 138 (112-168)
e
WGS + WTS (40 ICs) + prior ARSI
(Internal training)
log-rank: p = 0.034
Poor vs. Good Responders: q = 0.091
Ambiguous Prediction: 154 (133-NA)
Predicted - Good Responder: 166.5 (102-790)
Predicted - Poor Responder: 121 (103-NA)
Ensemble
(Internal training)
log-rank: p = 0.152
Poor vs. Good Responders: q = 0.421
Ambiguous Prediction: 166 (121-267)
Predicted - Good Responder: 133 (110-NA)
Predicted - Poor Responder: 119 (103-NA)
Cum. Ongoing Treatment
100%
75%
50%
25%
0%
0
500
1000
1500
2000
2500
No. at risk
Ambiguous Prediction
Predicted - Good Responder
Predicted - Poor Responder
Treatment duration (in days)

Suppl. Figure 7 - Survival analysis of predictors from the clinicogenomics models in the internal cohort.

- **a)** Survival analysis using overall survival from time of biopsy to death (event) using the three-group scheme of poor, good and ambiguous predictors for the clinicogenomics classification model on all included samples (*n* = 155). In total, 71 (46%) patients were censored (log rank *p* = 6.451934e-06 over all three groups, log-rank *q* between poor vs. good responders: 8.3e-06).
- **b)** Same as **a**, except for the internal training cohort (*n* = 79).
- **c)** Same as **a**, except for the internal validation cohort (*n* = 76).

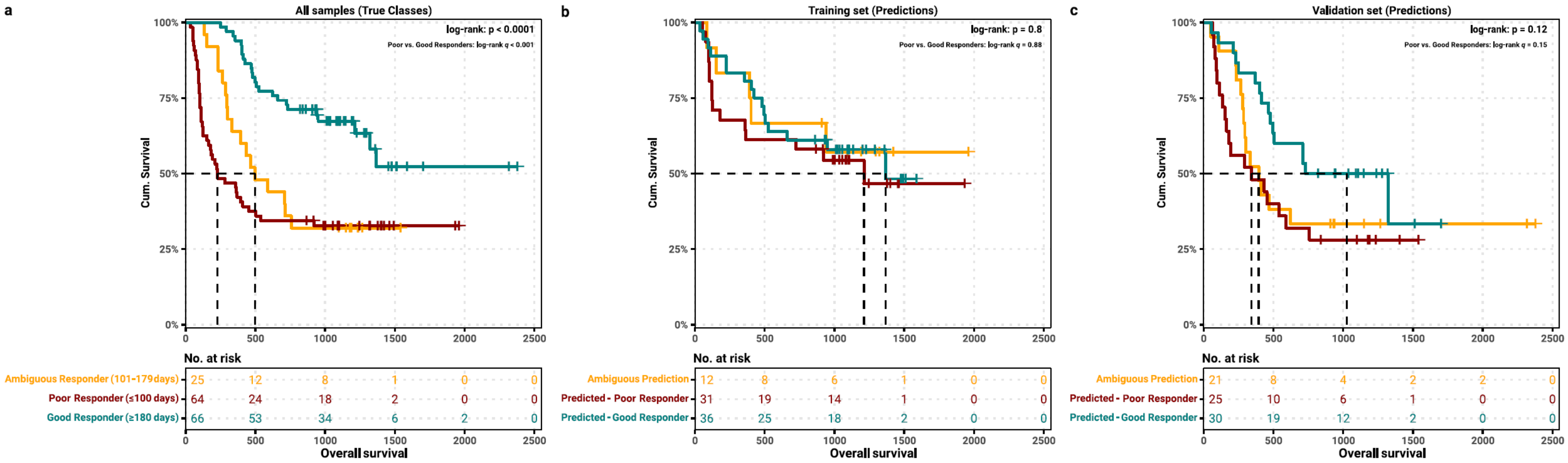

Suppl. Figure 8 - External validation genomics-only and transcriptomics-only model

Overall survival (OS) of patients in the external WCDT cohort from time of first biopsy to death for patients with an ARSI as the next therapy after biopsy. Survival curves were visualized using the Kaplan-Meier method and hazard ratios were calculated using Cox proportional hazards regression. P-value was calculated using the Wald-test/log-rank test. Median OS with 95% confidence intervals are shown per strata.Patients were sub-grouped based on **a)** the WGS-only model and **b)** the WTS (40 ICs)-only model, **c)** WTS + prior ARSI model, **d)** WGS + WTS + prior ARSI model and **e)** on the ensemble model.\\

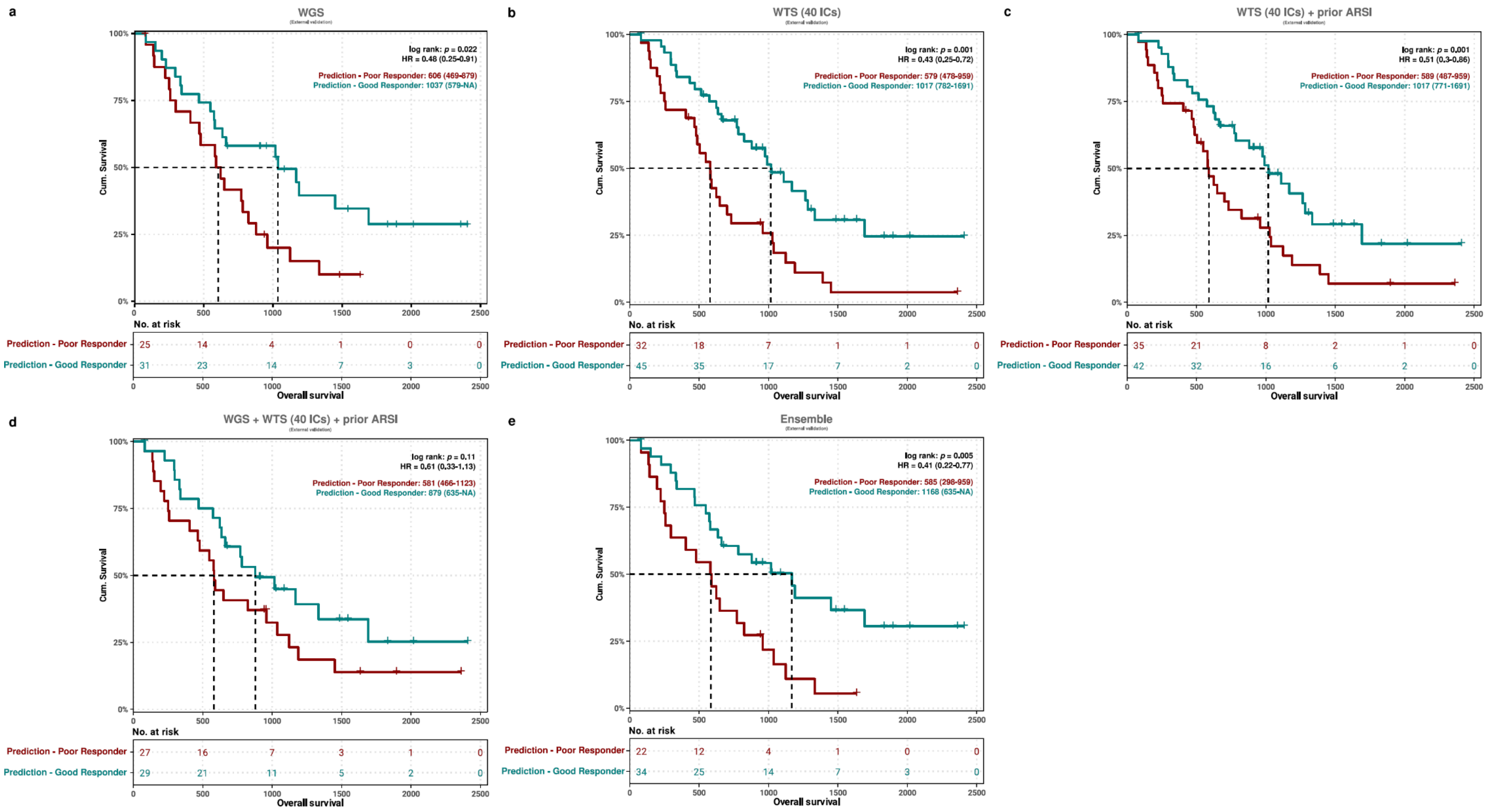

a
WGS
(External validation)
log rank: p = 0.022
HR = 0.48 (0.25-0.91)
Prediction - Poor Responder: 606 (469-879)
Prediction - Good Responder: 1037 (579-NA)
Cum. Survival
No. at risk
Prediction - Poor Responder 25 14 4 1 0 0
Prediction - Good Responder 31 23 14 7 3 0
Overall survival
b
WTS (40 ICs)
(External validation)
log rank: p = 0.001
HR = 0.43 (0.25-0.72)
Prediction - Poor Responder: 579 (478-959)
Prediction - Good Responder: 1017 (782-1691)
Cum. Survival
No. at risk
Prediction - Poor Responder 32 18 7 1 1 0
Prediction - Good Responder 45 35 17 7 2 0
Overall survival
c
WTS (40 ICs) + prior ARSI
(External validation)
log rank: p = 0.001
HR = 0.51 (0.3-0.86)
Prediction - Poor Responder: 589 (487-959)
Prediction - Good Responder: 1017 (771-1691)
Cum. Survival
No. at risk
Prediction - Poor Responder 35 21 8 2 1 0
Prediction - Good Responder 42 32 16 6 2 0
Overall survival
d
WGS + WTS (40 ICs) + prior ARSI
(External validation)
log rank: p = 0.11
HR = 0.61 (0.33-1.13)
Prediction - Poor Responder: 581 (466-1123)
Prediction - Good Responder: 879 (635-NA)
Cum. Survival
No. at risk
Prediction - Poor Responder 27 16 7 3 1 0
Prediction - Good Responder 29 21 11 5 2 0
Overall survival
e
Ensemble
(External validation)
log rank: p = 0.005
HR = 0.41 (0.22-0.77)
Prediction - Poor Responder: 585 (298-959)
Prediction - Good Responder: 1168 (635-NA)
Cum. Survival
No. at risk
Prediction - Poor Responder 22 12 4 1 0 0
Prediction - Good Responder 34 25 14 7 3 0
Overall survival

Suppl. Figure 9 - Importance of individual features to clinicogenomics model

Value of corresponding logistic regression model coefficients for all features of the clinicogenomics model (based on n = 79 (LOOCV folds) in each feature). Negative coefficient values mean that as the value of the given feature increases, the prediction probability also increases for the bad responder label. All feature coefficient values are negative, which can be explained by prevalence of disruptive SVs, overall higher tumor burden and prior ARSI treatment in patients who respond worse to therapy.

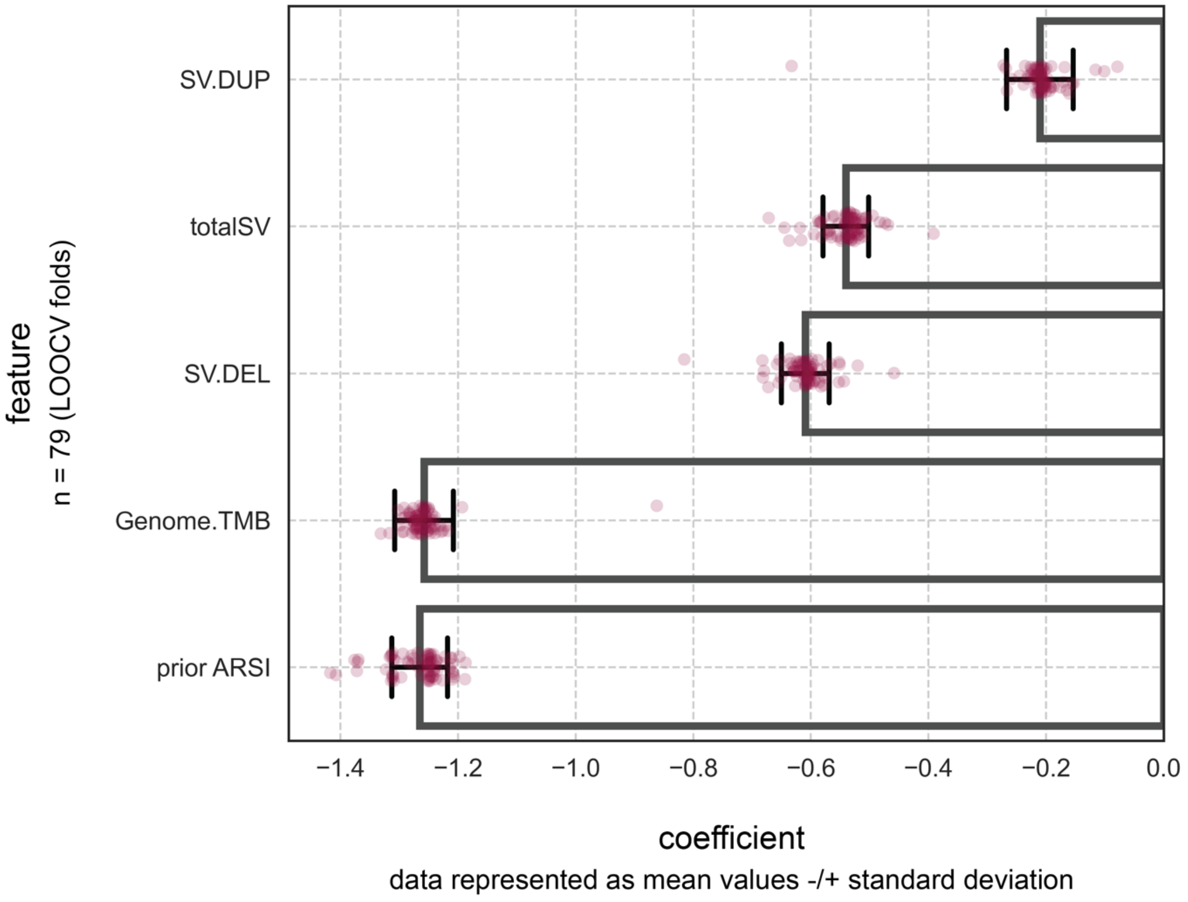

Suppl. Figure 10 - Distribution of individual genomic features in cohorts

Unscaled and standard scaled genomic feature distributions in the training, internal and external cohorts. TMB in the external cohort shows a wider skewed distribution compared to the training and internal cohort. Standard scaling of each dataset was performed prior to classification.

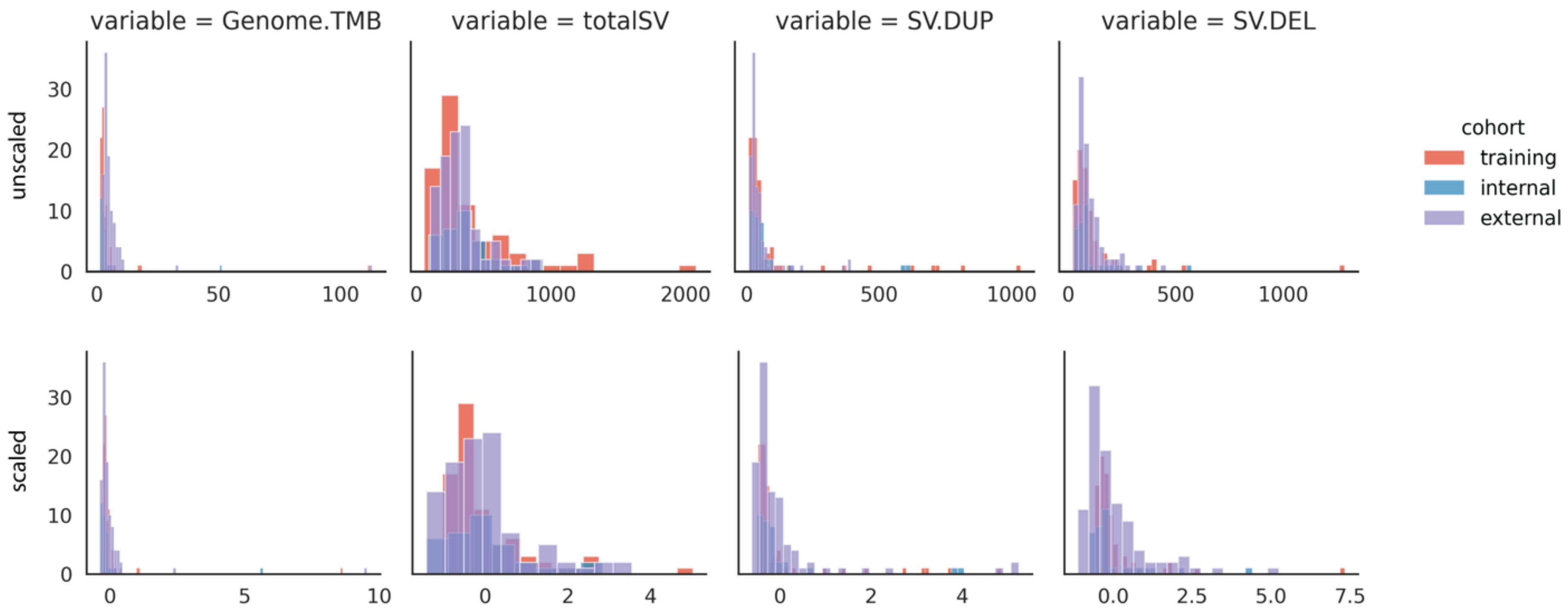

Suppl. Figure 11 - Evaluation of different dimensionality reduction techniques in LOOCV

ROC curves and AUC of a) sparse PCA b) PCA and c) Independent Component Analysis experiments. All models were tested with component numbers ranging from 10-50. The best performing model was chosen based on this comparison.

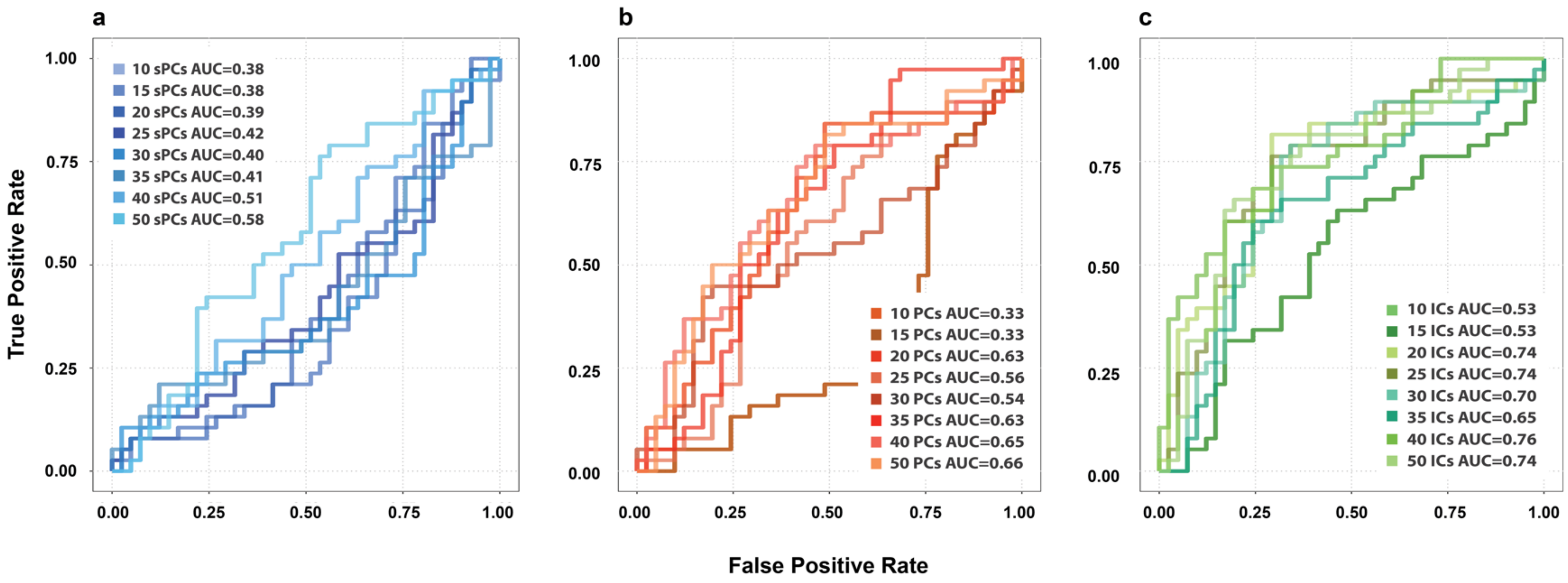

Suppl. Figure 12 - Comparison non-filtered and filtered transcriptomics model for external validation

For external validation of the transcriptomics model, certain genes were filtered out, as the used genome build versions differ between the training set and the external cohort (GENCODE v38 and GENCODE v28). To evaluate the potential effect of the missing genes on the classification, we compared the performance of a classifier that was trained on the full transcriptomics dataset with a classifier that was trained on the filtered transcriptomics dataset in the initial LOOCV step.

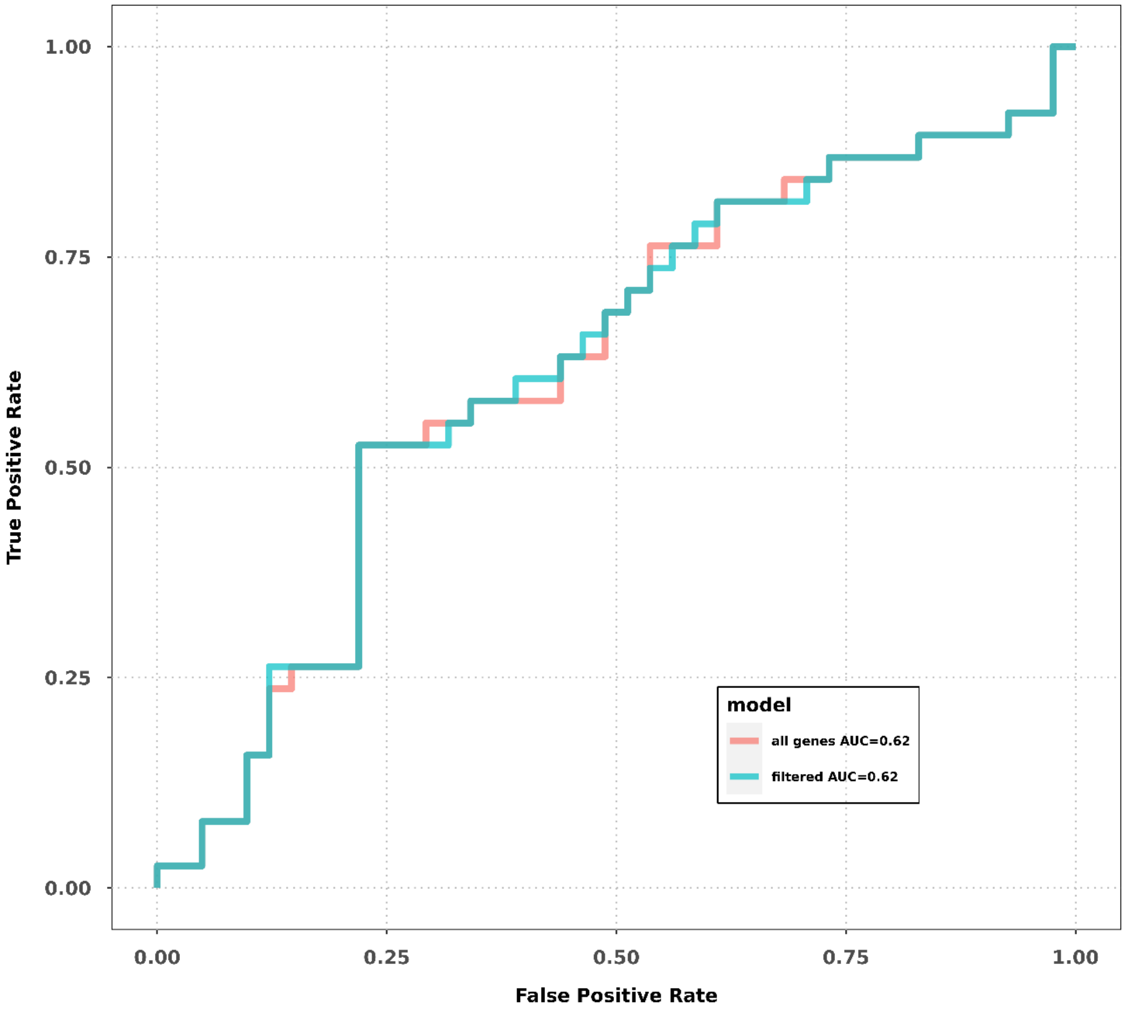

Supplementary Table Legends

Source Data - Overview of included patients and data presented in figures.

Overview of all data presented and quantified in this manuscript. Using the scripts and workflow described in “Code availability”, all figures can be reproduced.

(See Excel File)
