# Supplementary material for: Predicting response to enzalutamide and abiraterone in metastatic prostate cancer using whole-omics machine learning
Source: Nat Commun. 2023 Apr 8;14:1968. doi: 10.1038/s41467-023-37647-x (PMC10082805; doi:10.1038/s41467-023-37647-x)
Supplement: Supplementary file 3 — Reporting Summary [file 41467_2023_37647_MOESM3_ESM.pdf]

nature portfolio

Corresponding author(s): M.P. Lolkema
A.C. de Jong

Last updated by author(s): Mar 8, 2023

Reporting Summary

Nature Portfolio wishes to improve the reproducibility of the work that we publish. This form provides structure for consistency and transparency in reporting. For further information on Nature Portfolio policies, see our Editorial Policies and the Editorial Policy Checklist.

Statistics

For all statistical analyses, confirm that the following items are present in the figure legend, table legend, main text, or Methods section.

n/a	Confirmed	
☐	☒	The exact sample size (*n*) for each experimental group/condition, given as a discrete number and unit of measurement
☐	☒	A statement on whether measurements were taken from distinct samples or whether the same sample was measured repeatedly
☐	☒	The statistical test(s) used AND whether they are one- or two-sided<br>*Only common tests should be described solely by name; describe more complex techniques in the Methods section.*
☐	☒	A description of all covariates tested
☐	☒	A description of any assumptions or corrections, such as tests of normality and adjustment for multiple comparisons
☐	☒	A full description of the statistical parameters including central tendency (e.g. means) or other basic estimates (e.g. regression coefficient) AND variation (e.g. standard deviation) or associated estimates of uncertainty (e.g. confidence intervals)
☐	☒	For null hypothesis testing, the test statistic (e.g. *F*, *t*, *r*) with confidence intervals, effect sizes, degrees of freedom and *P* value noted<br>*Give P values as exact values whenever suitable.*
☒	☐	For Bayesian analysis, information on the choice of priors and Markov chain Monte Carlo settings
☒	☐	For hierarchical and complex designs, identification of the appropriate level for tests and full reporting of outcomes
☒	☐	Estimates of effect sizes (e.g. Cohen's *d*, Pearson's *r*), indicating how they were calculated

Our web collection on statistics for biologists contains articles on many of the points above.

Software and code

Policy information about availability of computer code

Data collection	No software used.
Data analysis	The initial workflows and software for the processing of the WGS data are available at https://github.com/hartwigmedical/. Any additional custom code and scripts used within this study (processing, analysis and visualization) have been deposited on Zenodo: DOI: 10.5281/zenodo.7712610<br>The custom R-based workflow (R2CPCT) used to further analyze the WGS-data obtained from HMF and CPCT-02 study is available on GitHub under the GPL-3.0 license: https://github.com/J0bbie/R2CPCT<br>The code used to further annotate genomic variants (as retrieved from HMF) using VEP is available on GitHub under the GPL-3.0 license: https://github.com/J0bbie/VariantAnnotation_VEP<br><br>List of packages used in the analysis:<br>precise=1.3 (PRECISE was directly installed from GitHub: https://github.com/NKI-CCB/PRECISE/tree/9f33ca78fb417a0337b6712f58ef2d83e1cb0e9f)<br>numpy=1.19.5<br>pandas=1.1.5<br>scikit-learn=0.24.1<br>scipy=1.5.4<br>DESeq2: 1.36.0<br>fgsea: 1.24.0<br>R2CPCT: 0.4

survminer: 0.4.9
gtsummary: 1.6.3

For manuscripts utilizing custom algorithms or software that are central to the research but not yet described in published literature, software must be made available to editors and reviewers. We strongly encourage code deposition in a community repository (e.g. GitHub). See the Nature Portfolio guidelines for submitting code & software for further information.

Data

Policy information about availability of data

All manuscripts must include a data availability statement. This statement should provide the following information, where applicable:

- Accession codes, unique identifiers, or web links for publicly available datasets
- A description of any restrictions on data availability
- For clinical datasets or third party data, please ensure that the statement adheres to our policy

- Discovery/internal validation cohort: CPCT-02 cohort with WGS, WTS and corresponding clinical data, available under restricted access and upon request via Hartwig Medical Foundation under accession code DR-071 (https://www.hartwigmedicalfoundation.nl/applying-for-data/)
- External validation cohort: WCDT cohort with WGS, WTS and corresponding clinical data of patients, who were treated directly after biopsy with ARSI and for who WGS and/or WTS was previously described by Quigley et al, Genomic Hallmarks and Structural Variation in Metastatic Prostate Cancer, Cell 2018. For a detailed description of data availability, we refer to this paper. Requests for data can be directed towards prof. dr. Felix Feng, E: Felix.Feng@ucsf.edu.

Human research participants

Policy information about studies involving human research participants and Sex and Gender in Research.

Reporting on sex and gender	Due to the nature of the disease (prostate cancer), this study was only performed in male.
Population characteristics	Metastatic castration resistant prostate cancer patients (mCRPC) in the Netherlands, with a mean age of 69 years, who were pretreated with 0-3 prior treatment lines for mCRPC.
Recruitment	Within 41 hospitals within the Netherlands, consecutive patients were recruited for the prospective CPCT-02 biopsy study. In short, patients were eligible if they had a locally advanced or metastatic solid tumor for which a new line of systemic treatment with a registered anti-cancer agent was indicated, and a safe tumor biopsy could be obtained. Patients could be recruited in every phase of mCRPC, independent of number of prior treatment lines and were asked for study participation before the start of a new treatment line. Biases could have been introduced by the willingness of patients to undergo a tissue biopsy, though, in general, biopsies could be obtained during a half-day visit within a few weeks after consent, and study participation did not significantly delay start of treatment.
Ethics oversight	The prospective CPCT-02 biopsy study (NCT01855477) has been approved by the medical ethical committee of the University Medical Center Utrecht and has been conducted in accordance with the Declaration of Helsinki.

Note that full information on the approval of the study protocol must also be provided in the manuscript.

Field-specific reporting

Please select the one below that is the best fit for your research. If you are not sure, read the appropriate sections before making your selection.

☒ Life sciences ☐ Behavioural & social sciences ☐ Ecological, evolutionary & environmental sciences

For a reference copy of the document with all sections, see nature.com/documents/nr-reporting-summary-flat.pdf

Life sciences study design

All studies must disclose on these points even when the disclosure is negative.

Sample size	No formal sample size calculation was performed, as the CPCT-02 study was designed to include patients with all kind of tumor types and treatments. As the number of patients in omics studies is in general always relatively small in comparison to the number of genomic features, we included all eligible mCRPC patients, who are treated with ARSI post-biopsy within the CPCT-02 study, in the current analyses.
Data exclusions	From 235 biopsies, 155 (66%) could be successfully analyzed by WGS. Eighty biopsies were excluded due to an unevaluable biopsy (n = 42), biopsy of the primary tumor (n = 13), whole exome sequencing instead of WGS (n = 11), protocol violation (n = 9), missing treatment information (n = 4) and a second evaluable biopsy in combination with ARSI within one patient (n = 1). The second evaluable biopsy of this patient was excluded to prevent overfitting in the analyses.
Replication	Due to the known high accuracy and depth of the sequencing, omics data were not replicated within this study. For model development, Leave One Out Cross Validation was performed to test the robustness of the results.

Randomization	As the main reason for stop of ARSI is progression of disease and rarely toxicity, patients were stratified according to treatment duration (TD) as surrogate for treatment response. Patients were stratified in good (TD ≥180 days), ambiguous (TD 101-179 days) and poor (TD ≤100 days) responders. Cut-off values were based on clinical practice. We considered patients with a treatment duration of ≤100 days as true poor responders, as 100 days (~12 weeks) is typically the first major decision point for treatment (dis)continuation according to the PCWG3 criteria. In addition, another threshold was set at ≥180 days to distinguish the true good responders from the ambiguous responders. To minimize the chance of bias due to incorrectly categorized patients, only the good and poor responder group were used for biomarker discovery and training of the classification model. Nevertheless, for a complete overview of the patient cohort, the ambiguous responders are visualized in the figures and are included during testing of the classification model.
Blinding	Not applicable, as we used both clinical outcome and omics data to develop prediction models. Predictions were performed after treatment of the patients and were not reported to the treating physician.

Reporting for specific materials, systems and methods

We require information from authors about some types of materials, experimental systems and methods used in many studies. Here, indicate whether each material, system or method listed is relevant to your study. If you are not sure if a list item applies to your research, read the appropriate section before selecting a response.

Materials & experimental systems

n/a	Involved in the study
☒	☐ Antibodies
☒	☐ Eukaryotic cell lines
☒	☐ Palaeontology and archaeology
☒	☐ Animals and other organisms
☐	☒ Clinical data
☒	☐ Dual use research of concern

Methods

n/a	Involved in the study
☒	☐ ChIP-seq
☒	☐ Flow cytometry
☒	☐ MRI-based neuroimaging

Clinical data

Policy information about clinical studies

All manuscripts should comply with the ICMJEguidelines for publication of clinical research and a completedCONSORT checklist must be included with all submissions.

Clinical trial registration	NCT01855477
Study protocol	The latest version of the full trial protocol will be attached to this form.
Data collection	Between February 2015 and October 2019, 235 patients with mCRPC were included within CPCT-02 in 41 hospitals in the Netherlands and treated with AAP or enzalutamide directly after a fresh-frozen biopsy. A list of participating hospitals is published on the website of CPCT (cpct.nl/ziekenhuizen). Two patients were included twice, resulting in the inclusion of 233 unique patients. Core needle biopsies were performed by an interventional radiologist according to local institutional guidelines during a half-day visit. Biopsy sites were chosen based on clinical judgement, safety and (prior) imaging. Subsequently, the freshly frozen biopsies were sequenced by the Hartwig Medical Foundation, Amsterdam, the Netherlands. From 235 biopsies, 155 (66%) could be successfully analyzed by WGS. Matched WTS data of the malignant tissue was available for 113 patients. As CPCT-02 is an ongoing study with more than 4000 patients, we used a snapshot of the clinical data from December 19th 2021 for the current analysis (ALEA Clinical). Clinical data collection is performed by trained local data managers and managed by a central data manager.
Outcomes	As the main reason for stop of ARSI is progression of disease and rarely toxicity, patients were stratified according to treatment duration (TD) as surrogate for treatment response. Patients were stratified in good (TD ≥180 days), ambiguous (TD 101-179 days) and poor (TD ≤100 days) responders. Cut-off values were based on clinical practice. We considered patients with a treatment duration of ≤100 days as true poor responders, as 100 days (~12 weeks) is typically the first major decision point for treatment (dis)continuation according to the PCWG3 criteria. In addition, another threshold was set at ≥180 days to distinguish the true good responders from the ambiguous responders. To minimize the chance of bias due to incorrectly categorized patients, only the good and poor responder group were used for biomarker discovery and training of the classification model. Nevertheless, for a complete overview of the patient cohort, the ambiguous responders are visualized in the figures and are included during testing of the classification model.<br><br>Classification models were validated in the external West Coast Dream Team cohort (WCDT), which included mCRPC patients treated with ARSI after biopsy. WGS was available for 56 patients, while WTS was available for 77 patients. Clinical outcome was defined as overall survival from time of biopsy to death of any cause.
